# Real-world multicenter study of rezvilutamide plus androgen deprivation therapy in Chinese patients with high-volume metastatic hormone-sensitive prostate cancer

**DOI:** 10.3389/fonc.2025.1657772

**Published:** 2025-10-01

**Authors:** Shuang Peng, Ruochen Zhang, Zijun Zou, Qichen Wei, Caixia Dai, Jinfeng Wu, Liefu Ye, Yongbao Wei, Li Huang

**Affiliations:** ^1^ Department of Urology, The Second Xiangya Hospital, Central South University, Changsha, China; ^2^ Shengli Clinical Medical College of Fujian Medical University, Department of Urology, Fujian Provincial Hospital, Fuzhou University Affiliated Provincial Hospital, Fuzhou, China; ^3^ Department of Urology, Guizhou Provincial People’s Hospital, Guiyang, China; ^4^ Department of Urology, Gutian County Hospital, Ningde, China; ^5^ Clinical Nursing Teaching and Research Section, The Second Xiangya Hospital of Central South University, Changsha, Hunan, China

**Keywords:** rezvilutamide, androgen deprivation therapy, high-volume metastatic hormone-sensitive prostate cancer, real-world study, PSA response

## Abstract

**Background:**

Rezvilutamide combined with androgen deprivation therapy (ADT) has become a first-line standard regimen for high-volume metastatic hormone-sensitive prostate cancer (mHSPC), but real-world evidence from China remains limited.

**Methods:**

This was a multicenter, retrospective real-world cohort study enrolling high-volume mHSPC patients diagnosed and treated with rezvilutamide plus ADT at multiple tertiary hospitals in China from August 2023 to March 2025. Baseline demographics, tumor burden, treatment regimen, and follow-up data were collected. The primary endpoints were 3-month PSA responses (PSA50, PSA90, PSA ≤0.2 ng/mL), subgroup efficacy, and adverse event rates. Multivariable logistic regression was used to identify independent predictors for achieving PSA ≤0.2 ng/mL at 3 months. Efficacy and safety outcomes were compared with major randomized controlled trials (RCTs) and other real-world studies.

**Results:**

A total of 236 patients with high-volume mHSPC were enrolled, with a median age of 74.9 years. The rates of achieving PSA50, PSA90, and PSA ≤0.2 ng/mL at 3 months were 99.15%, 88.98%, and 39.41%, respectively. Subgroup analysis showed consistent efficacy across age, ECOG, and Gleason strata, with only lower baseline PSA predicting higher likelihood of undetectable PSA. Multivariable analysis further indicated that baseline PSA showed a trend toward being an independent predictor for achieving PSA ≤0.2 ng/mL (P=0.063). The incidence of any-grade and grade 3–4 adverse events was 65% and 23%, respectively, with no new severe safety signals observed. All results were highly consistent with RCTs such as CHART.

**Conclusion:**

Rezvilutamide plus ADT provides rapid and profound PSA responses with a favorable safety profile in real-world Chinese patients with high-volume mHSPC. Baseline PSA serves as an important predictor for short-term biochemical response, supporting its clinical value as a standard first-line therapy.

## Introduction

Patients with high-volume metastatic hormone-sensitive prostate cancer (mHSPC) have a particularly poor prognosis. For a long time, androgen deprivation therapy (ADT) has been the mainstay of treatment; however, ADT alone offers limited disease control, is prone to progression to castration resistance, and yields poor long-term survival rates (1). In recent years, several large-scale randomized controlled trials (RCTs) have confirmed that combination therapies with novel androgen receptor (AR) inhibitors (such as abiraterone, enzalutamide, apalutamide) or chemotherapy significantly improve survival outcomes in mHSPC patients, and these regimens are now recommended as clinical standard treatments (2).

Rezvilutamide is a novel oral AR inhibitor characterized by low blood-brain barrier permeability (3). It has demonstrated potent antitumor activity and a favorable safety profile in hormone-sensitive prostate cancer (4, 5). The phase III CHART RCT confirmed that rezvilutamide combined with ADT significantly prolongs radiographic progression-free and overall survival in high-volume mHSPC patients, with manageable adverse events and a marked improvement in quality of life, establishing rezvilutamide plus ADT as a new standard of care for this population in China (4).

Although international and domestic RCTs and meta-analyses have established the efficacy of rezvilutamide (4, 6), real-world data in Chinese patients—particularly concerning PSA kinetics, stratified efficacy, and safety in high-volume disease—remain limited (5, 7). Previous studies have shown that PSA50, PSA90, and early undetectable PSA are dynamic surrogate markers closely correlated with survival and are sensitive indicators of efficacy (8). This study focuses on the real-world efficacy and safety of rezvilutamide plus ADT in Chinese multicenter high-volume mHSPC patients, with detailed analysis of PSA response kinetics, subgroup efficacy, and adverse event spectrum, and provides a comparison with mainstream RCT and prospective data to guide clinical practice.

## Patients and methods

### Patient population

This study included male patients diagnosed with high-volume mHSPC at urology or oncology departments of multiple tertiary hospitals in China between August 2023 and March 2025. All diagnoses conformed to international prostate cancer guidelines and CHAARTED criteria (4, 9): (1) pathologically or radiographically confirmed prostate cancer; (2) evidence of distant metastases meeting the definition of high-volume disease—at least four bone metastases with at least one outside the vertebral column and pelvis, or the presence of visceral metastases (such as lung, liver), regardless of the number of bone lesions.

#### Inclusion criteria

1. Male, aged ≥18 years;
2. Initial diagnosis of mHSPC, with no prior chemotherapy or next-generation AR inhibitor (e.g., abiraterone, enzalutamide); prior first-generation anti-androgen therapy (e.g., bicalutamide, flutamide) permitted if ≤3 months;
3. First systemic therapy was rezvilutamide plus ADT, with baseline assessment completed prior to initiation;
4. Complete clinical data available, including demographic, staging, pathology, PSA and imaging data, as well as efficacy and safety data during follow-up;
5. Written informed consent for scientific use of clinical data, or waiver as approved by the institutional ethics committee.

#### Exclusion criteria

1. Concomitant or prior other malignancy (except those cured for >5 years);
2. Concurrent enrollment in other anti-tumor drug clinical trials or interventional studies;
3. Severe organ dysfunction (heart, liver, kidney, etc.) during treatment that precludes continuation of ADT or rezvilutamide;
4. Incomplete follow-up or key data missing, making efficacy/safety evaluation impossible;
5. Other situations deemed unsuitable for enrollment by the investigator (e.g., poor compliance).

All cases were independently reviewed and confirmed by at least two senior physicians through electronic medical records and follow-up data to ensure accuracy and consistency. Definitions of high-volume disease, stratification criteria, and diagnostic procedures were consistent with internationally accepted guidelines and studies. All data collection and analysis adhered to the Declaration of Helsinki and subsequent amendments (10). The protocol was approved by the ethics committees of all participating centers, and informed consent was obtained from all enrolled patients.

### Data collection

Patient baseline demographics and clinical characteristics (including age, BMI, ECOG performance status, Gleason score, smoking, comorbidities, sites and number of metastases, baseline PSA, etc.), treatment regimens, and prior therapy history were extracted from electronic medical records and follow-up systems. Efficacy endpoints included baseline PSA, PSA nadir, time to nadir, and PSA responses at 3, 6, 9, and 12 months (≥50% reduction [PSA50], ≥90% reduction [PSA90], PSA ≤0.2 ng/mL) (8). Safety analysis covered the incidence of all-grade and grade 3–4 adverse events, as well as common adverse event types (e.g., fatigue, gastrointestinal symptoms, hematologic toxicity) (11).

### Statistical analysis

All statistical analyses were performed using IBM SPSS Statistics version 26.0 (IBM Corp., Armonk, NY, USA) and R version 4.3.0. Continuous variables were summarized as mean ± standard deviation or median (range) according to distribution as assessed by the Shapiro–Wilk test. Categorical variables were presented as counts and percentages. Between-group comparisons of continuous variables were conducted using independent-sample t-tests or Mann–Whitney U tests as appropriate; categorical variables were compared using Pearson’s chi-square test or Fisher’s exact test. For ordinal categorical variables (such as bone lesion count), the Cochran-Armitage trend test or logistic regression analysis was used to evaluate dose-response relationships or trends.

Efficacy endpoints (including rates of PSA50, PSA90, and PSA ≤0.2 ng/mL at 3 months) were summarized for the entire cohort and for predefined subgroups (age, ECOG, baseline PSA, Gleason score, visceral metastasis, bone lesion count). Differences in efficacy outcomes between subgroups were assessed using chi-square or Fisher’s exact tests, with trend tests for ordinal variables where applicable.

Additionally, to explore independent predictors of achieving PSA ≤0.2 ng/mL at 3 months, multivariable logistic regression analysis was performed. The dependent variable was achievement of PSA ≤0.2 ng/mL at 3 months. Covariates included age, BMI, ECOG performance status, Gleason score, baseline PSA, bone lesion count, presence of visceral metastasis, comorbidity count, smoking status, and prior treatment history. Odds ratios (ORs) and 95% confidence intervals (CIs) were calculated. Variables of clinical relevance or significance in univariate analysis were entered into the model. All tests were two-sided, with a P-value <0.05 considered statistically significant.

## Results

### Patient characteristics

A total of 236 patients with high-volume metastatic hormone-sensitive prostate cancer (mHSPC) were included in this multicenter retrospective study. The mean age was 74.9 ± 4.9 years, and the mean BMI was 24.05 ± 2.05 kg/m². ECOG performance status was 0 in 46.6% of patients, 1 in 38.6%, and 2 in 14.8%. Gleason score distribution was as follows: 17.4% (score 7), 34.3% (score 8), 41.9% (score 9), and 6.4% (score 10). A history of smoking was reported in 34.7% of patients, and 69.1% had at least one comorbidity (**Table 1**).

**Table 1:** Baseline characteristics of patients.

Variable	n (%) or Mean ± SD/Median (Range)
Age, years	74.9 ± 4.9
BMI	24.05 ± 2.05
ECOG Performance Status	0: 110 (46.6%)
	1: 91 (38.6%)
	2: 35 (14.8%)
Gleason Score	7: 41 (17.4%)
	8: 81 (34.3%)
	9: 99 (41.9%)
	10: 15 (6.4%)
Smoking Status	No: 154 (65.3%)
	Yes: 82 (34.7%)
Comorbidity Count	0: 73 (30.9%)
	1: 91 (38.6%)
	≥2: 72 (30.5%)
Bone Lesion Count	≤10: 42 (17.8%)
	11-20: 152 (64.4%)
	>20: 42 (17.8%)
Soft Tissue Metastasis	No: 168 (71.6%)
	Yes: 67 (28.4%)
Visceral Metastasis	No: 192 (81.4%)
	Yes: 44 (18.6%)
Prior Treatment	No: 172 (72.5%)
	Yes: 65 (27.5%)
Baseline PSA (ng/mL)	180.14 (12.93–314.28)

For metastatic burden, 17.8% of patients had ≤10 bone lesions, 64.4% had 11–20 lesions, and 17.8% had >20 lesions. Soft tissue metastasis was present in 28.4% of cases, and visceral metastasis in 18.6%. Prior systemic treatment before rezvilutamide plus ADT was given in 27.5% of patients. The median baseline PSA was 180.14 ng/mL (range, 12.93–314.28).

### Treatment efficacy

Following treatment with rezvilutamide plus androgen deprivation therapy, the median PSA nadir was 0.21 ng/mL (range, 0.00–0.40), and the median time to PSA nadir was 415.0 days (range, 169.3–624.1 days). At 3 months, 99.15% of patients achieved a PSA50 response (≥50% decline from baseline), 88.98% achieved a PSA90 response (≥90% decline), and 39.41% had PSA ≤0.2 ng/mL(**Table 2**). These findings demonstrate a robust early biochemical response in this high-volume cohort.

**Table 2:** Treatment efficacy outcomes.

Variable	Value
PSA Nadir (ng/mL), median (range)	0.20 (0.00–0.40)
Time to PSA Nadir (days), median (range)	415.0 (169.3–624.1)
3-month PSA50, n (%)	234 (99.15%)
3-month PSA90, n (%)	210 (88.98%)
3-month PSA ≤0.2 ng/mL, n (%)	93 (39.41%)

### Subgroup analysis

Subgroup analyses (**Table 3**) indicated no significant difference in PSA50 or PSA90 rates between most stratifications, including age (<75 vs. ≥75 years), ECOG status (0 vs. ≥1), and Gleason score (<9 vs. ≥9). Notably, patients with baseline PSA below the cohort median (<180.14 ng/mL) had a significantly higher rate of achieving PSA ≤0.2 ng/mL at 3 months compared to those with higher baseline PSA (48.3% vs. 30.5%, p=0.008) (**Table 3**). No significant difference was observed in PSA response rates between patients with and without visceral metastases or among bone lesion subgroups. Trend analysis for bone lesion number also showed no statistically significant association with biochemical response endpoints.

**Table 3:** Subgroup analysis of efficacy outcomes.

Subgroup	n	3m-PSA50 (%)	3m-PSA90 (%)	3m-PSA ≤ 0.2 (%)	PSA50 p value	PSA90 p value	PSA ≤ 0.2 p value
Age <75 years	104	99.00%	87.50%	41.30%			
Age ≥75 years	132	99.20%	90.20%	37.90%	1	0.662	0.684
ECOG 0	43	100.00%	90.70%	32.60%			
ECOG ≥1	193	99.00%	88.60%	40.90%	1	1	0.399
Baseline PSA <180.14 ng/mL	118	99.20%	84.70%	48.30%			
Baseline PSA ≥180.14 ng/mL	118	99.20%	93.20%	30.50%	1	0.061	0.008**
Gleason <9	119	98.30%	89.90%	39.50%			
Gleason ≥9	117	100.00%	88.00%	39.30%	0.498	0.8	1
No visceral metastasis	192	99.50%	88.50%	40.60%			
Visceral metastasis	44	97.70%	90.90%	34.10%	0.339	0.794	0.529
Bone lesion ≤10	42	100.00%	92.90%	45.20%			
Bone lesion 11–20	152	99.30%	88.20%	40.80%			
Bone lesion >20	42	97.60%	88.10%	28.60%			
Trend test for bone lesion (p)					0.25	0.486	0.12

P values (e.g., <0.05) are marked with ** to indicate statistical significance. Other p values represent results from trend tests or group comparisons using chi-square or Fisher’s exact tests.

### Multivariable analysis: independent predictors of achieving PSA ≤0.2 ng/mL at 3 months

Multivariable logistic regression analysis was performed to further evaluate independent predictors of achieving undetectable PSA (≤0.2 ng/mL) at 3 months. Baseline PSA demonstrated a trend toward being an independent predictor of achieving PSA ≤0.2 ng/mL (OR=0.997, 95% CI: 0.994–1.000, P=0.063), suggesting that lower baseline PSA levels may be associated with a higher likelihood of achieving undetectable PSA at 3 months. Other variables, including age, ECOG performance status, Gleason score, bone lesion count, presence of visceral metastasis, comorbidity count, smoking status, and prior treatment, did not show statistically significant associations after adjustment (all P > 0.05) (**Table 4**). These findings were consistent with the results of the univariate subgroup analysis.

**Table 4:** Multivariable logistic regression analysis of factors associated with achieving PSA ≤0.2 ng/mL at 3 months.

Factor	OR	P value	95% CI
Age	0.99	0.744	0.94, 1.05
BMI	0.97	0.689	0.85, 1.11
ECOG 1 vs 0	1.32	0.455	0.63, 2.77
ECOG 2 vs 0	1.5	0.405	0.57, 3.94
Gleason ≥9	0.97	0.917	0.56, 1.69
Baseline PSA	0.997	0.063	0.994, 1.000
Bone metastasis 11–20 vs ≤10	0.77	0.48	0.38, 1.58
Bone metastasis >20 vs ≤10	0.45	0.091	0.18, 1.14
Visceral metastasis	0.82	0.582	0.40, 1.67
Number of comorbidities	1.05	0.772	0.77, 1.43
Smoking history	1.57	0.118	0.89, 2.78
Prior treatment	1.3	0.399	0.71, 2.40

### Safety and adverse events

Among the safety population, 65% of patients experienced at least one adverse event (AE) of any grade, and 23% experienced grade 3–4 AEs (**Table 5**). The most common AEs included fatigue (17.3%), decreased appetite (13.1%), hypertension (12.3%), diarrhea (10.1%), nausea or vomiting (7.6%), rash (6.3%), anemia (5.5%), elevated liver enzymes (5.1%), hyperglycemia (4.7%), and neutropenia (4.2%). No unexpected safety signals were observed, and the regimen was well tolerated overall.

**Table 5:** Adverse events.

Adverse event type	n (%)
Any AE (≥Grade 1)	154 (65.0%)
Grade 3–4 AE	55 (23.0%)
Fatigue	41 (17.3%)
Decreased appetite	31 (13.1%)
Hypertension	29 (12.3%)
Diarrhea	24 (10.1%)
Nausea/Vomiting	18 (7.6%)
Rash	15 (6.3%)
Anemia	13 (5.5%)
Liver enzyme elevation	12 (5.1%)
Hyperglycemia	11 (4.7%)
Neutropenia	10 (4.2%)

## Discussion

This multicenter real-world study systematically analyzed the efficacy and safety of rezvilutamide combined with ADT in a Chinese cohort with high-volume mHSPC. The rates of achieving PSA decline closely aligning with the results of the CHART study (4, 8, 12). These findings indicate that rezvilutamide plus ADT produces a rapid and profound biochemical response in patients with high-volume disease. Subgroup analyses indicated that clinical characteristics such as age, ECOG performance status, and Gleason score had limited influence on short-term PSA response. Only patients with lower baseline PSA experienced deeper PSA reductions (P=0.008), consistent with previous reports.

Comparison with other real-world studies, our short-term biochemical outcomes are broadly concordant with prior evidence. Specifically, the 3-month PSA50, PSA90, and PSA ≤0.2 ng/mL rates observed in our high-volume cohort (99.15%, 88.98%, and 39.41%) closely mirror those reported for the high-volume subgroup in the CHART trial (99.0%, 89.0%, and 39.2%) (4, 8), suggesting that the effectiveness of rezvilutamide plus ADT in real-world practice is comparable to that observed under trial conditions. Notably, compared to a recent real-world multicenter study of low-volume mHSPC patients in China (TAU 2025) (5), the short-term PSA response rates in our high-volume group are similar or slightly higher, but the low-volume cohort showed a further increase in deep PSA remission rates over time, reaching 100%, 92%, and 83% at 12 months. This indicates that early PSA kinetics are broadly comparable across disease volumes, but deep and sustained biochemical responses tend to be more pronounced in low-volume disease. Differences in disease burden, follow-up duration, and study design may also account for these variations.

Furthermore, our multivariate analysis demonstrated that baseline PSA showed a trend toward being an independent predictor of achieving undetectable PSA at 3 months. Traditional prognostic factors such as age, ECOG status, Gleason score, number of bone metastases, and presence of visceral metastases were not significant predictors in high-volume patients, which is consistent with the findings in low-volume patients. This highlights baseline PSA as the most robust predictor of early biochemical response, regardless of disease burden.

In terms of safety, the rates of any-grade and grade 3–4 adverse events (65% and 23%) were also in line with previous RCT and real-world reports (5, 12), with no new severe safety signals observed. This consistency further supports that the tolerability profile of rezvilutamide plus ADT in everyday practice is comparable to that reported in controlled trial settings.

To our knowledge, this is the first large-scale, multicenter real-world analysis in Chinese high-volume mHSPC patients to systematically report short-term PSA kinetics, subgroup efficacy, and predictive factors, thus providing practical and evidence-based guidance for first-line treatment optimization in this setting. Further multivariable logistic regression analysis demonstrated that baseline PSA showed a trend toward being an independent predictor of achieving PSA ≤0.2 ng/mL at 3 months (OR=0.997, 95% CI: 0.994–1.000, P=0.063), indicating that patients with lower baseline PSA tended to have a higher likelihood of achieving a profound clinical response. Other traditional clinical factors such as age, ECOG score, Gleason score, bone metastasis count, and presence of visceral metastasis did not reach statistical significance after multivariate adjustment. This is consistent with the results of univariate and subgroup analyses and highlights the unique value of baseline PSA in predicting short-term efficacy.

Regarding adverse events, 65% of patients experienced at least one adverse event (any grade), and the incidence of grade 3–4 adverse events was 23%. The most common events were fatigue, decreased appetite, and hypertension, which is largely consistent with the safety profile of second-generation AR inhibitors observed in the CHART (4), ARCHES (13), and TITAN (14) studies. No new severe safety signals or significant central nervous system adverse effects were observed, which is consistent with the low blood-brain barrier permeability of rezvilutamide (3).

In recent years, novel endocrine therapies and multimodal combination strategies have continued to develop, and both doublet (ADT + next-generation AR inhibitor or abiraterone/docetaxel) and triplet (ADT + next-generation AR inhibitor + docetaxel) regimens have been widely recommended by international guidelines (2, 15). Multiple RCTs, such as LATITUDE, ARCHES, TITAN, and ENZAMET, have confirmed that abiraterone, enzalutamide, and apalutamide combined with ADT significantly improve survival in patients with high-volume mHSPC, while ARASENS and PEACE-1 further suggest that triplet therapy may provide additional benefits for certain high-risk populations (2, 15). However, triplet regimens are associated with increased toxicity and higher discontinuation rates, so individualized decision-making is required based on patient-specific factors (16).

Our real-world data demonstrate that rezvilutamide plus ADT achieves PSA response rates and safety outcomes comparable to those reported in major RCTs, while maintaining favorable quality of life and symptom control. This provides a valuable treatment option for high-volume patients who are unable to tolerate chemotherapy or prefer to minimize toxicity (5, 12). Compared indirectly with similar next-generation AR inhibitors (such as enzalutamide and apalutamide), rezvilutamide shows comparable or even superior safety in terms of biochemical response and quality of life (6, 17). However, from a health-economic perspective, a recent Chinese pharmacoeconomic analysis found that, at current prices, rezvilutamide was less cost-effective than bicalutamide in high-volume mHSPC, but would become cost-effective if the price were reduced by at least 10% or further incorporated into the NRDL (National Reimbursement Drug List) (7). However, due to differences in study inclusion criteria, follow-up duration, and efficacy assessment systems, it is currently difficult to draw definitive conclusions regarding relative superiority (18).

There are several limitations to this study. First, as a retrospective cohort, some baseline information or follow-up data may be missing or subject to bias. Second, the follow-up period was relatively short, so long-term survival outcomes and late adverse events could not be evaluated. We focused on 3-month PSA responses because early PSA kinetics (PSA50, PSA90, and PSA ≤0.2 ng/mL) have been validated in multiple RCTs as surrogate markers for long-term outcomes. Nonetheless, extended follow-up is necessary to confirm the association between early biochemical response and durable survival benefits. Third, this study lacked a comparator arm (e.g., ADT alone or ADT combined with other next-generation AR inhibitors), which significantly limits the interpretability of treatment efficacy. Therefore, our results should be interpreted with caution and validated in prospective controlled trials. Finally, variations in practice patterns, patient compliance, and adjunctive therapies among centers may also have influenced the results.

## Conclusion

In summary, multicenter real-world data demonstrate that rezvilutamide combined with ADT achieves rapid and profound PSA responses in Chinese patients with high-volume metastatic hormone-sensitive prostate cancer, with favorable safety and quality-of-life outcomes. Its efficacy is highly consistent with international RCT results, providing an effective and well-tolerated treatment option for high-risk patients, particularly those unable to tolerate chemotherapy. However, considering the limitations of this study, these conclusions need to be further validated in larger, long-term, and prospective studies to optimize treatment strategies and survival outcomes for patients with high-volume disease.

## Data Availability

The original contributions presented in the study are included in the article/supplementary material. Further inquiries can be directed to the corresponding author.
